# Long-term functional prognosis of patients with aneurysmal subarachnoid hemorrhage treated with rehabilitation combined with hyperbaric oxygen: Case-series study

**DOI:** 10.1097/MD.0000000000018748

**Published:** 2020-01-17

**Authors:** Yong Wang, Yali Gao, Minjie Lu, Yuewei Liu

**Affiliations:** Rehabilitation Medicine Center, Fuxing Hospital, Capital Medical University, Beijing, China.

**Keywords:** aSAH, functional prognosis, hyperbaric oxygen, predictive factors, rehabilitation

## Abstract

The long-term prognosis of patients with aneurysmal subarachnoid hemorrhage (aSAH) has received increasing attention in recent years. Hyperbaric oxygen and rehabilitation are already used in clinical treatment of patients with aSAH, but it is unclear whether it can improve the long-term prognosis of patients postoperation. The purpose of this study was to evaluate the long-term prognosis and prognostic factors associated with combined rehabilitation and hyperbaric oxygen therapy for patients with aSAH.

Information were retrospectively collected from patients with aSAH treated from October 2014 to July 2017, including demographics, history of hypertension, Hunt–Hess Grade at the time of onset, location of aneurysm, surgical treatment, status of delayed cerebral ischemia and tracheotomy, level of consciousness impairment (Glasgow Coma scale [GCS], neurologic function damage (National Institutes of Health Stroke Scale [NIHSS]), status of hydrocephalus, time of initial hyperbaric oxygen and rehabilitation therapy, as well as duration and frequency of hyperbaric oxygen therapy, and so on. Long-term functional prognosis was measured by modified Rankin scale (mRS), and mRS ≤3 was defined as good prognosis. Univariate and multivariate logistic regression were used to analyze predictors associated with poor prognosis.

A total of 44 patients with aSAH were enrolled, and 25 patients (56.8%) had a good functional prognosis 6 months after disease onset. Univariate analysis showed age (*P* = .028), hyperbaric oxygen and rehabilitation start time (*P* = .039), NIHSS (*P* = .000), hydrocephalus (*P* = .024), frequency of hyperbaric oxygen therapy (*P* = .016), GCS ≤8 points (*P* = .000), and tracheotomy (*P* = .007) were associated with prognosis. Multivariate logistic regression analysis showed that only a higher NIHSS score was an independent predictor of poor prognosis (odds ratio = 1.59; 95% confidence interval, 1.10–2.30).

More than 50% of patients with aSAH can achieve a good functional prognosis after combined rehabilitation and hyperbaric oxygen therapy. The severity of neurological impairment before treatment is closely related to poor prognosis.

## 1 Introduction

Aneurysmal subarachnoid hemorrhage (aSAH) is a cerebrovascular disease that seriously endangers public health, accounting for 85% of spontaneous subarachnoid hemorrhage and 3% to 5% of stroke. About half of patients’ age was less than 55 years old.^[1,2]^ The annual incidence of aSAH worldwide is about 2 to 16/100,000 people.^[3]^ Despite the rapid development in treatment approaches, such as surgery, interventional embolization, and intensive care in recent years, the early mortality rate of aSAH is still high. Epidemiological studies have shown that the average mortality rate of aSAH is 27% to 44%.^[4]^ Many survivors also live with dysfunctions in cognition and exercise, and more than 40% of patients with aSAH are unable to return to pre-morbid conditions. Therefore, improving long-term prognosis in patients with aSAH remains critical.^[5]^

Although it is generally believed that early rehabilitation in the acute phase of neurological injury is effective, there is no consensus on aSAH. The main concern is that early rehabilitation can increase the risk of intracranial pressure and cerebral vasospasm, leading to secondary brain damage.^[6,7]^ Recent studies have shown that early rehabilitation of aSAH patients is safe and feasible.^[7,8]^ However, whether early rehabilitation can improve the long-term prognosis of patients with aSAH is still unknown. Karic et al showed that early rehabilitation does not improve the long-term function of patients with aSAH.^[9]^ Cerebral vasospasm and delayed cerebral ischemia are the main causes of aSAH-related disability and death. Hyperbaric oxygen therapy (HBOT) can inhibit cerebral vasospasm by regulating protein kinase pathway and inflammatory cytokine secretion. In addition, HBOT can also reduce the infarct size and improve neurological function by inhibiting oxidative stress, reducing brain edema, increasing oxygen carrying capacity, promoting blood–brain barrier integrity and increasing cerebral blood flow.^[10,11]^ However, due to the lack of access to hyperbaric oxygen chambers, the clinical application of HBOT is limited. Meanwhile, study has shown that the time window indicated for HBOT treatment is short and delayed HBOT may aggravate cerebral ischemic injury. Therefore, the efficacy of HBOT in the treatment of aSAH remains controversial.^[12]^ Factors such as advanced age, female, hypertension, Hunt–Hess Grade, delayed cerebral ischemia, craniotomy, hydrocephalus, persistent consciousness impairment, and severe infection are all associated with poor prognosis of aSAH,^[13,14]^ but it remains poorly understood what factors can affect the effectiveness of HBOT and rehabilitation.

To the best of our knowledge, there have been no reports on the study of patients with aSAH after early rehabilitation combined with HBOT. The objective of this study was to evaluate whether HBOT combined rehabilitation can improve functional outcomes 6 months after aSAH diagnosis and analyze predictors associated with poor prognosis.

## 2 Methods and materials

### 2.1 Study design and subjects

This is a single-center retrospective study that included eligible patients who underwent aSAH treatment at the Rehabilitation Center of the Fuxing Hospital affiliated to Capital Medical University (Beijing, China) from October 2014 to July 2017. Inclusion criteria were: surgically treated adult aSAH patients (age ≥18 years). Exclusion criteria were: untreated ruptured aneurysm; previous history of SAH, cranio-cerebral trauma or neurodegenerative disease; hemodynamic instability; middle ear barotrauma; chronic otitis media; late end of life. The study was approved by the Institute Review Board of Fuxing hospital.

### 2.2 Clinical data

The following information were collected from hospital's electronic medical record system for each patient: age, gender, site of ruptured aneurysm (anterior communicating artery, posterior communicating artery, middle cerebral artery and other location), aSAH classification, surgical procedure (clipping, embolization, clipping + embolization), decompressive craniectomy, delayed cerebral ischemia (DCI), history of hypertension, degree of neurological damage before treatment, consciousness status, HBOT and rehabilitation treatment start time, tracheotomy, hydrocephalus and HBOT frequency. The classification of aSAH was based on the Hunt–Hess Grade, with I–III being the low-grade, and IV–V the high-grade aSAH.^[15]^ The degree of neurological impairment was classified using the National Institutes of Health Stroke Scale (NIHSS) and NIHSS ≥11 was classified as moderate to severe neurological impairment.^[16]^ The state of consciousness is based on the Glasgow Coma scale (GCS), and GCS ≤8 is classified into severe coma.^[17]^ DCI is defined as a reduction in focal neurological impairment or a reduction of GCS score by at least 2 points, which excludes other causes.^[14]^

### 2.3 HBOT treatment

In the 2.0 atmosphere absolute environment, 100% oxygen was taken for 60 minutes, separated by 5 minutes of the cabin air once daily. The compression time was 20 minutes, and the decompression time was 25 minutes.

### 2.4 Rehabilitation treatment

The entire rehabilitation treatment was completed by a team consisting of cross-disciplinary members, including rehabilitation physician, physical therapist (PT), occupational therapist, speech therapist, and nurse. The rehabilitation program was comprehensive and included the following components: mobilization, resting in neutral position during rest and sleep, passive exercises for contracture prevention, pulmonary rehabilitation, guidance on daily living activities including swallowing and eating, cognitive rehabilitation, speech therapy, and balance training. The main component of the rehabilitation program was developed based on the activity program established by Olkowski et al for aSAH patients.^[8]^ According to the specific needs of each patient, the activity program was built based upon the step-by-step mobility training. The core of this stepped program is to get off the bed for sitting, standing, and walking training as soon as possible, even if the patient was still in the state of consciousness impairment, severe neurological damage, and tracheotomy. The mobility protocols mainly consisted of 6 stages: head of bead, bed sitting, chair and PT bed sitting, bedside and PT bed standing, assisted step and walking training (Table 1).

**Table 1:**
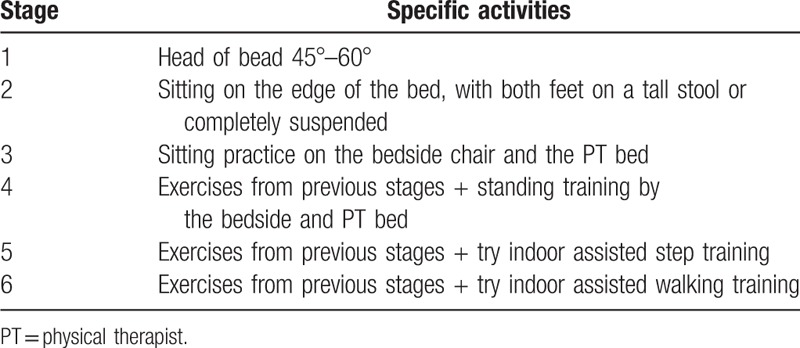
Progressive activity training program.

Each stage requires completion of at least 2 to 3 times, each time 15 to 30 minutes. If activity was fully tolerated at 1 stage, the patient progressed to the next stage the following day. The criteria for complete tolerance were as follows: monitoring indicators within the normal range, no neurological deterioration and no complaints of discomfort. The monitoring indicators were: respiratory rate <40 beats/min, mean arterial pressure <110 mm Hg, 50 beats/min > heart rate < 150 beats/min, SaO2 decreased <5%, no new arrhythmia such as atrial fibrillation or ventricular tachycardia. In addition to the above activities, other rehabilitation treatments included: swallowing function assessment and training, speech function assessment and training, guided activity of daily living training, passive maintenance rang of joint training for patients with consciousness and neurological impairment, and placement of functional limbs on the bed, as well as muscle strength training, and so on. The daily rehabilitation content of each patient, the monitoring of tolerance indicators, and whether to enter the next stage were discussed and decided by the rehabilitation team members.

### 2.5 Statistical analysis

Statistical analysis was performed using SPSS19.0 software. The continuous data were expressed as mean ± standard deviation, and analyzed by independent-samples *t* test. The categorical data were expressed by frequency, analyzed by Chi-square tests or Fisher exact tests. Multivariate logistic regression was performed for the factors with *P* < .05 in the univariate analysis, to determine the independent predictors of efficacy. *P* < .05 indicated statistically significant difference.

## 3 Results

### 3.1 Study population

A total of 44 patients with aSAH were enrolled, including 16 males and 28 females, aged 37–80 (57.0 ± 11.6) years. There were 39 cases of single aneurysm and 5 cases of multiple aneurysms. The ruptured aneurysm was located at anterior communicating artery in 16 cases, posterior communicating artery in 13 cases, middle cerebral artery in 10 cases, internal carotid artery in 3 cases, and vertebral basilar artery in the remaining 2 cases. As for Hunt-Hess grade, there were 10 cases of grade II, 11 cases of grade III, 23 cases of grade IV. Surgical treatment included 18 cases of simple embolization, 23 cases of simple clipping, 3 cases of clipping + embolization, and 9 cases of decompressive craniectomy. There were 10 cases of delayed cerebral ischemia, 9 cases of hydrocephalus, and 29 cases with a history of hypertension. Hyperbaric oxygen and rehabilitation therapy were initiated 8 to 76 (34.5 ± 17.2) days from the onset. The mean NIHSS score was 10.5 ± 8.7 (ranged 1–31), of which 21 patients had moderate to severe neurological impairment. As for the damage of consciousness: there were 13 patients with GCS ≤8 points. There were 16 patients with tracheotomy, and 13 of them had the gas-cut sleeve eventually removed. The total number of hyperbaric oxygen treatments was 8 to 70 (28.3 ± 17.9) times. At the end of 6-month follow-up: mRS was ≤3 points in 25 cases, of which 12 of the 25 high-grade aSAH cases recovered well.

### 3.2 Univariate analysis of prognostic factors

Patients were divided into a good prognosis (mRS ≤3) and a poor prognosis (mRS >3) group. Univariate analysis showed significant difference between the 2 groups in age (*P* = .028), hyperbaric oxygen and rehabilitation start time (*P* = .039), NIHSS (*P* = .000), hydrocephalus (*P* = .024), frequency of hyperbaric oxygen (*P* = .016), GCS ≤8 (*P* = .000), and tracheotomy (*P* = .007) (Table 2).

**Table 2:**
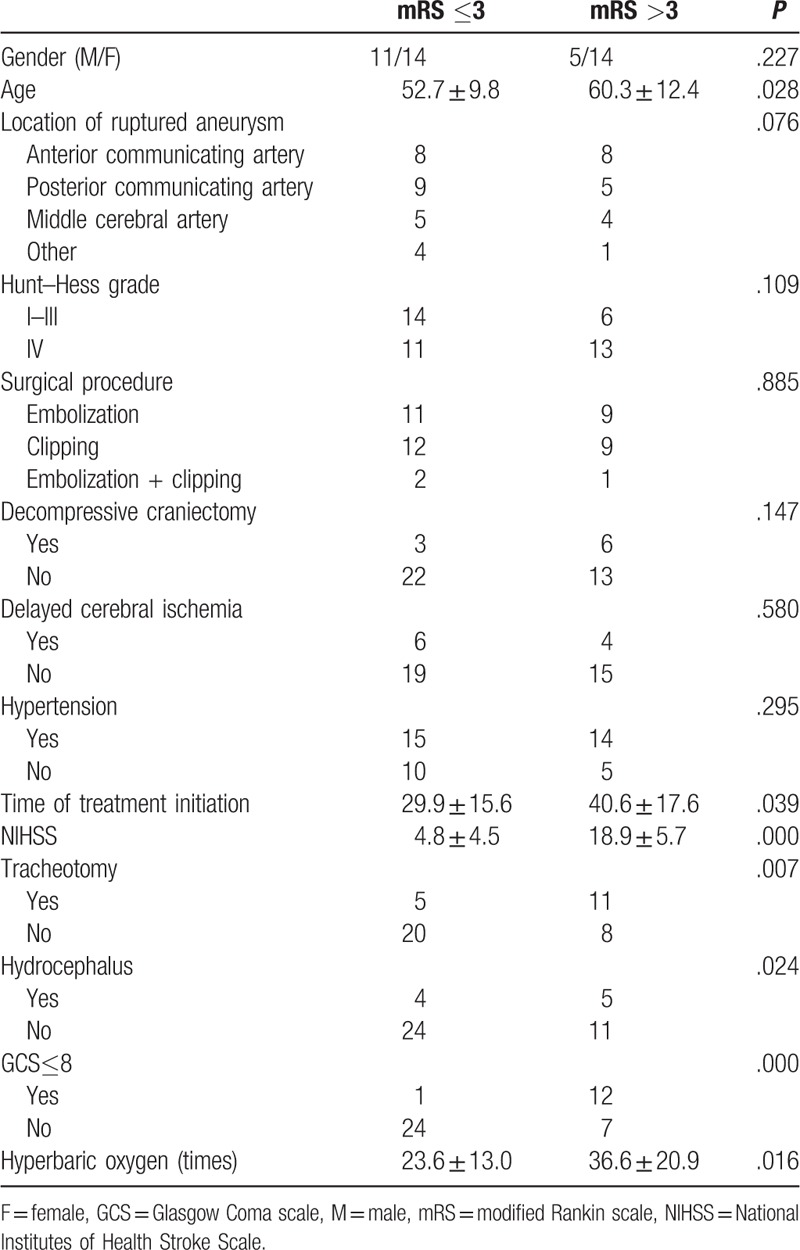
Univariate analysis of prognostic factors.

### 3.3 Multivariate logistic regression analysis of patients’ prognosis

Multivariate logistic regression analysis was performed with age, HBOT and rehabilitation start time, NIHSS, hydrocephalus, HBOT frequency, GCS ≤8 points, and tracheotomy as independent variables. The final model showed that NIHSS (odd rates = 1.59; 95% confidence interval, 1.10–2.30) was an independent risk factor for poor prognosis (*P* = .013) (Table 3).

**Table 3:**
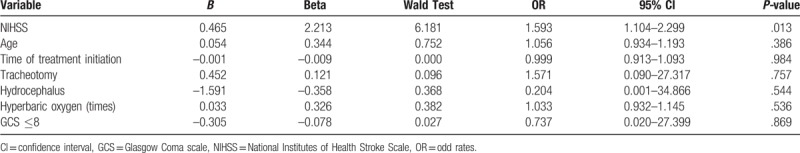
Multivariate logistic regression analysis of prognostic factors.

## 4 Discussion

The main finding of our study is that HBOT combined with personalized rehabilitation program can improve the prognosis of patients with aSAH at 6-month after the disease onset. The more severe the neurological function damage, the worse the prognosis would be.

In this study, women, middle-aged and patients with single aneurysms accounted for the majority of patients. Ruptured aneurysms were mainly located at the anterior communicating artery, posterior communicating artery and middle cerebral artery, which was consistent with previous literature reports.^[1,2,18]^ The most important feature of our patients was that high-grade aSAH accounts for 52.3%, which is different from several recent studies of the early rehabilitation in patients with low-grade aSAH.^[6–9]^ According to the H-H grade, IV is defined as coma, moderate to severe hemiplegia and early denervation, and grade V is defined as deep coma, decerebrate rigidity, and sudden death.^[15]^ So, high-grade aSAH is closely related to poor prognosis.^[13,19]^ In addition, 29.5% of patients who were admitted to our department for HBOT and rehabilitation treatment were in a severe coma, 47.7% were in moderate to severe neurological impairment and 36.4% underwent tracheotomy.

In our study, 22.7% of patients had delayed cerebral ischemia after surgery. Delayed cerebral ischemia is currently recognized as one of the factors leading to poor prognosis in patients with aSAH.^[2,14]^ Cerebral vasospasm is one of the most important causes of delayed cerebral ischemia. Despite the use of various methods to prevent cerebral vasospasm, there are still 17% to 21% of aSAH patients with delayed cerebral ischemia.^[11,20]^ HBOT is to let patients breathe in 100% oxygen in an environment above 1 atmosphere, which can double the level of physical dissolved oxygen in plasma, thus significantly improving the oxygen carrying capacity of blood. The increase in oxygen-carrying capacity can improve the function of damaged cell membranes, restore the balance of intracellular electrolytes, and improve the energy metabolism around the ischemic region. These may be the Mechanism for the treatment of delayed cerebral ischemia by HBOT.^[21]^

However, most of the current research on HBOT treatment of SAH is experimental, and clinical research is still limited. An RCT study by Tang XP found that HBOT could improve the functional outcome of patients with anterior circulation aSAH at 6 months after clipping procedure. They also found that HBOT could reduce middle cerebral artery flow rate and incidence of symptomatic cerebral vasospasm 7- and 14-days postoperation compared with the control group. The incidence of cerebral infarction and edema at 7, 14, and 21 days after surgery were also lower.^[22]^ However, unlike that study which started HBOT within 3 days after the surgery, the shortest time to start HBOT in our study was 8 days, and the longest was 76 days. Efrati et al found that HBOT can improve the neurological function of patients with chronic stroke (6–36 months of onset), and they believe that HBOT can still activate neuro-remodeling during the chronic phase of stroke.^[23]^ We also believe that HBOT can still improve the neurological function of aSAH patients after surgery and during the rehabilitation period. The therapeutic mechanism will still require further research in the future to confirm. Rehabilitation in early stage of stroke can reduce mortality, reduce complications, and improve long-term functional outcomes.^[24]^ Although aSAH is a subtype of stroke, its clinical features and pathophysiological mechanisms are different from those of other stroke subtypes, leading to significant differences in response to rehabilitation in patients with aSAH. On one hand, subarachnoid hemorrhage leads to diffuse injury more similar to severe traumatic brain injury, so in early stage aSAH patients often need surgery and stay in the intensive care unit (ICU) longer for monitoring and treatment. Prevention of various complications is the focus in this period. On the other hand, aSAH patients are younger, have fewer underlying diseases and are more likely to recover, so they generally respond better to rehabilitation than other types of stroke patients.^[8,25]^

Most of our patients were transferred from the Neurological ICU, and have been in bedridden for a while. Therefore, the rehabilitation program we adopted was mainly body position conversion activity, from the lying position to the sitting position, to the standing position and walking. For patients without altered consciousness or severe physical dysfunction, we shortened the time of stages 1 to 3 and started bedside assisted standing and walking training as soon as possible. For patients with severe consciousness impairment or physical dysfunction, early standing and walking is more difficult. Besides the therapist's assistance, we also used knee-ankle-foot joint orthoses to assist in standing and walking training, because leaving the bed early and starting standing activities not only improve neurological function,^[6]^ but also stimulate the recovery of consciousness by stimulating the reticular activation system.^[26]^ Several studies using similar rehabilitation programs have also shown that early rehabilitation can improve level of consciousness, promote neurological recovery, and improve long-term outcomes.^[9,13,25]^ More than half of the patients recovered well 6 months after HBOT combined rehabilitation, 48% of them recovered well from high-grade aSAH, and 81.3% of patients had the tracheotomy removed at discharge. In a study of aSAH patients who were still in a state of severely altered consciousness during rehabilitation, only 4.8% of patients recovered to functional independence and were able to eat by mouth 6 months after rehabilitation, and only 23.8% of patients eventually had tracheotomy removed.^[13]^

Factors such as advanced age, female, hypertension, Hunt-Hess classification, delayed cerebral ischemia, craniotomy, hydrocephalus, persistent disturbance of consciousness, and severe infection are all associated with poor prognosis.^[13,14]^ Multivariate regression analysis showed that only a higher NIHSS score at the time of rehabilitation was an independent predictor of poor outcomes after HBOT and rehabilitation in aSAH patients. The severity of neurological impairment in stroke patients is closely related to the prognosis of motor and cognitive function.^[27]^ At the same time, severe neurological damage can lead to many complications such as heart, lung, digestive tract and neuropsychological complications.^[16]^ These complications can affect the progress of HBOT and rehabilitation, especially the rehabilitation program based on body position conversion, thus affects the treatment outcome. Although GCS scores and tracheotomy were not independent predictors of poor prognosis, they significantly increased the score of NIHSS. In this study, only 1 of 13 patients with GCS recovered well, and only 5 of 16 patients undergone tracheotomy recovered well. Hydrocephalus was not an independent predictor, which may be related to the hydrocephalus shunt surgery undergone by our patients. Although there were no significant statistical differences, we found that the poor prognosis group started HBOT and rehabilitation in a later time, and required more HBOT treatment sessions, indicating that severe aSAH patients need to stay longer in the ICU and need more rehabilitation sessions.

There are several limitations in this study. First, it is a retrospective study of small sample of patients. Heterogeneity in the severity of the disease and the time of starting HBOT and rehabilitation might have an impact on the results of the study. Secondly, the time to evaluate the prognosis in this study was 6 months after onset, unlike more than 12 months follow up in other studies,^[9,19,28]^ which may underestimate the significant functional improvement in some patients during longer follow-up. In addition, aSAH often leads to cognitive dysfunction and affects the long-term prognosis of patients, but mRS is not sensitive enough to cognitive function, so we need to be careful when interpreting the results of this study.

## 5 Conclusions

We found that after combined HBOT and progressive rehabilitation based on postural conversion, 56.8% of patients with aSAH had a good functional outcome after 6 months of onset. The more severe the neurological damage at the start of HBOT, the worse the treatment outcome becomes.

## Author contributions

**Data curation:** Minjie Lu.

**Project administration:** Yali Gao.

**Resources:** Yali Gao, Yuewei Liu.

**Software:** Yali Gao.

**Writing – original draft:** Yong Wang.
